# Tofacitinib for Hospitalized Acute Severe Ulcerative Colitis Management (The TRIUMPH Study)

**DOI:** 10.1093/crocol/otaf013

**Published:** 2025-02-15

**Authors:** Neeraj Narula, Cara Pray, Hasan Hamam, Farhad Peerani, Tawnya Hansen, Talat Bessissow, Brian Bressler, Arathi Arun, Maria Schmit, Jane Castelli, John K Marshall

**Affiliations:** Department of Medicine, Division of Gastroenterology, McMaster University Medical Centre, Hamilton, ON, Canada; Department of Medicine, Division of Gastroenterology, McMaster University Medical Centre, Hamilton, ON, Canada; Department of Medicine, Division of Gastroenterology, McMaster University Medical Centre, Hamilton, ON, Canada; Division of Gastroenterology, University of Alberta Hospital, Edmonton, AB, Canada; Department of Medicine, Division of Gastroenterology, Health Sciences Centre/University of Manitoba, Winnipeg, MB, Canada; Division of Gastroenterology, McGill University Health Center, Montreal, QC, Canada; Department of Medicine, Division of Gastroenterology, St. Paul’s Hospital/University of British Colombia, Vancouver, BC, Canada; Department of Medicine, Division of Gastroenterology, McMaster University Medical Centre, Hamilton, ON, Canada; Department of Medicine, Division of Gastroenterology, St. Paul’s Hospital/University of British Colombia, Vancouver, BC, Canada; Department of Medicine, Division of Gastroenterology, McMaster University Medical Centre, Hamilton, ON, Canada; Department of Medicine, Division of Gastroenterology, McMaster University Medical Centre, Hamilton, ON, Canada

**Keywords:** acute severe ulcerative colitis, hospitalized ulcerative colitis, tofacitinib, Xeljanz, MTWSI, Lichtiger Index, patient-reported outcomes

## Abstract

**Background:**

Tofacitinib is a rapidly acting Janus kinase (JAK) inhibitor with increasing evidence of effectiveness in patients with acute severe ulcerative colitis (ASUC). However, there are scarce prospective data analyzing the efficacy and rapidity of action in hospitalized ASUC.

**Methods:**

The TRIUMPH study is a prospective open-label interventional trial of tofacitinib in hospitalized patients with ASUC conducted in 5 hospitals across Canada (Clinicaltrials.gov: NCT04925973). Eligible participants included biologic-naïve and experienced patients with ASUC refractory to 3 days of intravenous (IV) corticosteroids (Modified Truelove-Witts Severity Index [MTWSI] > 10 despite steroids). Participants were treated with tofacitinib 10 mg twice daily and assessed daily while in hospital. The primary outcome was day 7 clinical response (MTWSI reduction of > 3 from baseline and ≤ 10).

**Results:**

Among 24 subjects, 33.3% (8/24) had previous anti-TNF failure. Day 7 clinical response was achieved in 58.3% (14/24). The mean number of days to achieve clinical response was 2.4 (SD 1.3). Marked reduction in C-reactive protein was observed in responders within the first two days after tofacitinib initiation compared to nonresponders. Colectomy occurred in 25% (6/24) by 6 months, with no additional colectomy beyond this time point. Five participants reported a total of 13 adverse events.

**Conclusions:**

Tofacitinib is an effective rescue therapy in hospitalized patients with steroid-refractory ASUC. Randomized controlled trials are warranted to compare JAK inhibitors with other rescue therapies, including infliximab in steroid-refractory ASUC (Clinicaltrials.gov: NCT04925973).

## Graphical Abstract

**Graphical Abstract:**
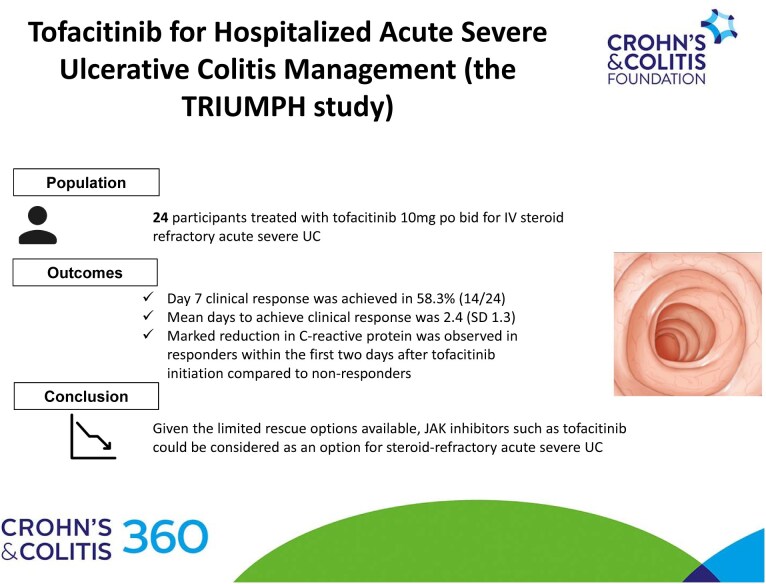

**Key Points**

- What is already known?
  JAK inhibitors such as tofacitinib have rapid onset of action in moderate-to-severe ulcerative colitis (UC)
- What is new here?
  Tofacitinib demonstrated efficacy for steroid-refractory acute severe UC with symptomatic and biochemical improvement demonstrated by day 2.
- How can this study help patient care?
  Given the limited rescue options available, JAK inhibitors such as tofacitinib could be considered as an option for steroid-refractory acute severe UC.

## Introduction

Acute severe ulcerative colitis (ASUC) will impact up to 25% of individuals with ulcerative colitis (UC).^1^ This potentially life-threatening condition requires hospital admission and timely initiation of rescue therapy to prevent deleterious outcomes such as toxic megacolon, colonic perforation, and the need for emergent colectomy. Intravenous (IV) corticosteroids are established as the foundation of ASUC management. However, up to 40% of patients will be refractory to corticosteroids and require additional rescue management strategies.^2,3^ Currently, both cyclosporin and infliximab are recognized as medical rescue therapies and have been studied in randomized controlled trials for ASUC. These trials have not demonstrated superiority of one of these therapies over the other, thus current clinical guidelines suggest either as a reasonable management option.^4–7^ Despite these rescue options, colectomy is still too often required, with estimated rates of 20%, 40%, and 60% at first, second and third admission, respectively.^1,8^

Tofacitinib is an oral small-molecule Janus kinase (JAK) inhibitor currently approved for induction and maintenance of moderate-to-severe UC based on the phase 3 clinical trial program OCTAVE.^9^ JAK inhibition downregulates signaling and production of several proinflammatory cytokines. It is also hypothesized that in ASUC, JAK inhibition paired with corticosteroids improves steroid responsiveness and decreases the need for additional rescue therapy.^10^ Post hoc analyses from the OCTAVE studies suggest that symptoms improve significantly as early as 3 days postinitiation of tofacitinib in comparison to placebo.^11^ Owing to its rapid onset of action, tofacitinib has been considered as a potential third option for medical rescue therapy in ASUC.

This open-label prospective interventional trial was performed to determine if there is benefit from tofacitinib in the hospitalized ASUC population who experience inadequate response to steroids.

## Methods

### Study Design

This was a multicenter, open-label prospective trial conducted across 5 Canadian hospitals. This study was conducted in compliance with the Declaration of Helsinki and was approved by each of the participating institutions’ research ethics boards. It was registered with the United States National Library of Medicine (Clinicaltrials.gov: NCT04925973). All participants provided written informed consent.

### Patient Population

Adults aged 18-75 with a known history or new diagnosis of UC hospitalized with ASUC were enrolled. Inclusion into the study required symptoms defined by Modified Truelove-Witts Severity Index (MTWSI) >10 despite a minimum of 3 days of IV corticosteroids at a dose equivalent to prednisone 50mg daily. Patients with a history of primary nonresponse or secondary loss of response to anti-TNFα/anti-integrin therapies/anti-interleukin therapies were permitted, with no recent adjustment in dosing prior to screening. Treatment with concomitant 5-ASA therapies was permitted. Once enrolled into the study, participants’ biologic and/or immunomodulator therapies were discontinued prior to initiation of tofacitinib. Those who had exposure to tofacitinib within 3 months prior to screening were excluded. Individuals with active enteric infections or clinical signs of sepsis were excluded. Additionally, patients with an indication for surgery (toxic megacolon, massive exsanguination, or perforation), current malignancy, serious comorbidity including immunodeficiency, myocardial infarction, or stroke within a month of enrollment, history of heart, respiratory, renal, hepatic failure, current abscess, or opportunistic infection were precluded from enrollment. Lastly, women with positive blood (beta-HCG) pregnancy test, currently lactating, or women of childbearing potential who were unwilling to use double barrier contraception were excluded.

### Procedures

Eligible patients underwent a flexible sigmoidoscopy at study entry to grade endoscopic severity. Participants were then started on tofacitinib 10 mg twice daily. All participants received pharmacologic thrombosis prophylaxis while in hospital. Participants were assessed daily using MTWSI, partial Mayo scores, and serum C-reactive protein (CRP). If after day 3 participants had an increase in MTWSI of >4 points, or an increase in CRP > 30 mg/L, they were given the opportunity to exit the study and offered an alternative medical therapy or surgery as deemed appropriate by their medical team.

By day 7 of treatment with tofacitinib, the primary outcome of clinical response was determined. Treatment responders were those with a decrease in MTWSI score ≥3 points from baseline and MTWSI ≤10 for 2 consecutive days. Those who responded to tofacitinib on or before day 7 were switched from intravenous corticosteroids to oral prednisone 40 mg daily and were followed as an inpatient for an additional 1-2 days to ensure they did not have rebound worsening of symptoms in hospital. Participants who continued to have clinical response were discharged on oral tofacitinib 10 mg twice daily and prednisone 40 mg daily with plan for fixed taper of prednisone by 5 mg per week. If participants required escalation of steroid dosing due to symptomatic worsening during their taper, they were considered treatment failures. After 8 weeks of tofacitinib 10 mg twice daily, maintenance dosing was offered per investigator discretion at 5 mg or 10 mg twice daily.

Following discharge from hospital, responders were assessed clinically at weeks 6, 12, 26, and 52. Biochemical monitoring was performed every 8 weeks and repeat endoscopic assessment took place at week 26. Adverse events were assessed for at each clinical follow-up.

Participants that did not respond to tofacitinib by day 7 were offered either infliximab or colectomy as deemed appropriate by the admitting physician. Furthermore, individuals who relapsed during study protocol were considered as failures of the study and underwent study procedures, including flexible sigmoidoscopy and bloodwork at the time of withdrawal.

### Definition of Response

Clinical response was defined as MTWSI reduction by 3 or more points, and MTWSI ≤ 10. Clinical remission was defined as partial Mayo score ≤2 with no subscore >1. Steroid-free clinical remission was defined as partial Mayo score ≤2 with no subscore >1 and free of corticosteroid use for ≥ 90 days. Endoscopic improvement was considered an endoscopic Mayo score of 0 or 1.

### Outcomes

The primary outcome was clinical response at day 7. Secondary outcomes included clinical response and remission at weeks 12, 26, and 52, colectomy and corticosteroid-free clinical remission at weeks 26 and 52, and endoscopic improvement at week 26. Additional outcomes included time to symptom improvement and biochemical improvement, and adverse events among participants treated with tofacitinib in the trial.

### Statistical Analysis

Descriptive statistics were used and presented as means and standard deviations or medians and interquartile ranges (IQR). Proportions of participants achieving the different remission and response outcomes were compared between responders and nonresponders with use of the Chi-squared test.

Univariate analyses were conducted to examine for baseline variables that were associated with achievement of day 7 clinical response. A one-way repeated measures ANOVA was used to assess differences in the evolution of biomarkers over time among day 7 responders and nonresponders. Lines of best fit were plotted and slopes compared using *t*-tests comparing interaction terms in the model.^12^ For all analyses, a *P*-value < .05 indicated statistical significance.

## Results

### Participant Characteristics

Baseline characteristics of the 24 steroid-refractory patients hospitalized for ASUC who were recruited to receive tofacitinib as rescue therapy are provided in Table 1. The mean age of participants was 42.5 years (SD 17.3). The majority of participants had a history of pan-ulcerative colitis and 75% (*n* = 18) had a Mayo Endoscopic subscore of 3 at study entry. One-third (*n* = 8) of participants had at least one previous biologic failure, and all of these participants had a history of anti-TNF use. No patients had previously been exposed to tofacitinib. At enrollment, the mean total Mayo Score was 10.1 (SD 1.4). Biochemical parameters reflected a severe UC population, including mean baseline CRP of 37.8 mg/L (SD 46.4), mean hemoglobin of 90.2 g/L (SD 31.1), and mean albumin of 28.5 g/L (SD 5.3). The median time for treatment with intravenous corticosteroids was 3.1 days (SD 0.4).

**Table 1.** Baseline characteristics.

**Total number of patients**	24
**Age,** mean (SD)	42.5 (17.3)
**Sex,** *n* (%)	
Male	13 (54.2)
Female	11 (45.8)
**Disease location,** *n* (%)	
Left-sided	2 (8.3)
Extensive	4 (16.7)
Pancolitis	18 (75)
**Disease duration,** years (IQR)	3 (6)
**Smoking status,** *n* (%)	
Nonsmoker	22 (91.7)
Ex-smoker	2 (8.3)
**Previous Biologics,** *n* (%)	8 (33.3)
Anti-TNF	8 (33.3)
Vedolizumab	2 (8.3)
Ustekinumab	2 (8.3)
**Labs**	
Hgb g/L, mean (SD)	90.2 (31.1)
CRP mg/L, median (IQR)	37.8 (46.4)
Fecal Calprotectin mcg/g, median (IQR)	2641 (4159.5)
Albumin g/L, mean (SD)	28.5 (5.3)
**Mayo Endoscope subscore 3,** *n* (%)	18 (75)
**Mayo Score,** mean (SD)	
Total	10.1 (1.4)
Stool frequency	2.8 (0.5)
Rectal bleeding	1.8 (0.8)
Physician global assessment	2.8 (0.4)
Endoscopic score	2.8 (0.4)
**Mean time of treatment with intravenous corticosteroids,** days (SD)	3.1 (0.4)

### Efficacy Outcomes

The primary outcome of clinical response by day 7 was achieved in 14 of 24 (58.3%) of patients (Table 2). At day 7, 18 individuals remained on tofacitinib, and 5 participants (20.8%) had achieved clinical remission. The mean number of days to achieve a clinically significant response was 2.43 (SD 1.28). In the 14 participants that responded, improvement of diarrhea subscore to 0 or 1 was achieved by over 60% of individuals by day 3 (Supplementary Figure 1), and by day 4 almost 90% of responders had a subscore of 0 or 1 for visible blood in stool (Supplementary Figure 2). Among responders, the last participant who demonstrated a clinical response did so by day 5. Among subjects who did not attain clinical response by day 5, continuing treatment on days 6 and 7 did not lead to a subsequent clinical response.

**Table 2.** Percentage of patients who achieved clinical response or remission at day 7, week 12, 26, and 52.

	Day 7	Week 12	Week 26	Week 52
Total number of patients who were still taking drug at the timepoint	18	11	11	9
Clinical response %, (*n*)	58.3 (14/24)	37.5 (9/24)	33.3 (8/24)	33.3 (8/24)
Clinical remission %, (*n*)	20.8 (5/24)	37.5 (9/24)	33.3 (8/24)	29.2 (7/24)
Endoscopic improvement %, (*n*)	N/A	N/A	33.3 (8/24)	N/A
Steroid-free clinical remission %, (*n*)	0	N/A	33.3 (8/24)	29.2 (7/24)

Clinical response (MTWSI reduction 3 or more points and MTWSI ≤ 10).

Clinical remission (Partial Mayo score ≤ 2, with no subscore > 1).

Steroid-free (no corticosteroid use for ≥ 90 days).

Endoscopic improvement (endoscopic Mayo score 0 or 1).

**Figure 1.**
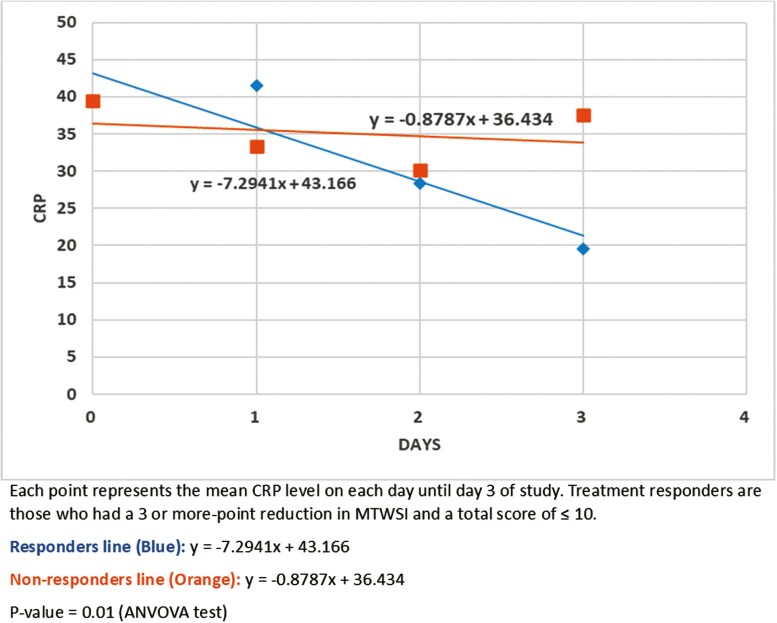
Daily mean CRP trends for tofacitinib responders vs nonresponders.

Figure 1 presents the trends in mean CRP levels through the first 4 days of treatment in responders and nonresponders to tofacitinib. Responders experienced a significant decrease in mean CRP (F(2, 11), *P* = .01) compared to nonresponders based on one-way repeated measures ANOVA, as the slope of the line of best fit for nonresponders was almost flat (−0.8787). Similarly, a significantly greater decrease in median CRP (F(2, 5), *P* = .048) in responders compared to nonresponders was observed based on one-way repeated measures ANOVA (Supplementary Figure 3).

At week 12, 9 of 11 individuals who continued on tofacitinib (37.5%) achieved clinical response, clinical remission, and corticosteroid-free remission. At week 26, 11 persisted on therapy, among who 8 (33.3%) achieved clinical response, clinical remission, steroid-free clinical remission, and endoscopic improvement. Lastly at week 52, there remained 9 participants in the trial, 8 (33.3%) who had achieved clinical response and 7 (29.2%) were in clinical remission and corticosteroid-free clinical remission.

Treatment failures over the course of the trial are summarized in Table 3. The majority of failures (10/24) were within the first 7 days of enrollment. Four patients had a lack of response, 4 required colectomy, one withdrew from the study one day after starting, and one experienced a serious adverse event and discontinued therapy. Among participants who had lack of response, 3 were treated with in-hospital infliximab induction (2 of whom clinically responded) and one was treated with upadacitinib due to prior infliximab exposure (and achieved clinical response). Between days 7 and 90, 5 additional participants had loss of response, including 2 participants that required colectomy, one at week 6 and one at week 8. Twelve participants completed the 8 weeks of induction at 10mg bid and were able to wean off steroids completely. Six of the participants continued 10mg bid for maintenance, while the other 6 were transitioned to 5mg twice daily. Of the 6 individuals who were transitioned to a lower maintenance dose, 3 relapsed. All 3 individuals who relapsed were dose escalated to 10mg twice daily and clinical response was recaptured in 2 participants. All 6 colectomies that occurred were due to refractory disease.

**Table 3.** Patient disposition over the course of the trial.

	Tofacitinib (*n* = 24)
**Absence of clinical response at day 7**	10
Withdrawal	1
Colectomy	4
Lack of response	4
Adverse event	1
**Failure after day 7 and before day 90**	5
Withdrawal	0
Colectomy	2
Relapse	3
Adverse event	0
**Failure after day 90 and before day 180**	0
Withdrawal	0
Colectomy	0
Relapse	0
Adverse event	0
**Failure after day 180**	1
Withdrawal	1
Colectomy	0
Relapse	0
Adverse event	0
Total treatment failure	16

Supplementary Table 1 outlines baseline characteristics of tofacitinib responders and nonresponders, with the exclusion of one individual who withdrew from the study after one day of treatment. No baseline variable on univariate analysis was predictive of day 7 clinical response, including previous biologic use or albumin level (Supplementary Table 2).

### Safety Outcomes

A total of 5 participants (21%) experienced a total of 13 adverse events throughout the study period (Supplementary Table 3). Many of the treatment-related adverse events were mild, including leukocytosis, headache, tachycardia, nausea, and decreased appetite. One patient experienced a serious adverse event. This individual was 37 years of age with multiple comorbidities, including HIV treated with emtricitabine/tenofovir disoproxil fumarate with undetectable viral load and cocaine use prior to hospital admission, who on day 3 of the trial was experiencing hemorrhagic shock and had a syncopal event and ischemic stroke. They were subsequently transferred to ICU for cardiovascular support. The neurologist and cardiologist who assessed the patient in-hospital did not feel that stroke was due to use of tofacitinib.

## Discussion

This open-label prospective interventional trial demonstrated that tofacitinib is an effective rescue therapy in patients hospitalized with ASUC refractory to IV steroids. Overall, 58.3% of participants treated with tofacitinib 10mg twice daily responded by day 7, and in some cases improvement of clinical and biochemical parameters, including diarrhea, hematochezia, and CRP was observed as early as 2 days after tofacitinib initiation. At week 52, 9/24 individuals remained on tofacitinib with the majority of these individuals achieving persistent steroid-free clinical remission and endoscopic improvement. A total of 25% of participants underwent colectomy over the duration of the trial.

Up to 40% of individuals with ASUC will not clinically respond to IV corticosteroids and will require second-line rescue strategies.^2,3,13^ Infliximab and cyclosporin are the only 2 additional rescue therapies supported by international guidelines and consensus statements. In our analysis, tofacitinib responders achieved a clinically significant response in 2.43 days (SD 1.28), comparable to the median response times reported for infliximab and cyclosporin of 4-5 days.^4^ Moreover, CySIF reported 7-day clinical response rates for infliximab and cyclosporin of 84% and 85%, respectively, which was numerically higher than the 58% observed in this trial.^4^ However, it is important to note CySIF included only bio-naïve patients, whereas 33.3% of participants in the TRIUMPH trial were bio-experienced.^4^ In contrast, the PREDICT-UC study observed 7-day clinical response rates with infliximab of 61% and 65% for patients treated with 5 mg/kg and 10 mg/kg, respectively.^14^ The TACOS trial demonstrated a high clinical response rate with tofacitinib in ASUC of 83%, although the individuals in this analysis were treated with 10 mg 3 times daily of tofacitinib for 7 days and were not nonresponders of IV steroids.^10^ Sustained clinical response has been a limitation beyond acute rescue therapy. In CySIF, by day 98, 31/57 (54%) failed infliximab and 35/58 (60%) failed cyclosporin.^4^ Our trial showed a numerically similar 3-month treatment failure rate of 15/24 (62.5%), despite one-third of the participants having failed prior biologic therapies.

Tofacitinib has been demonstrated as an effective add-on therapy to corticosteroids. It is hypothesized that populations of individuals with corticosteroid nonresponsive ASUC have higher expression of proinflammatory cytokines leading to decreased cellular sensitivity to steroids. Through JAK/STAT inhibition, it is postulated that tofacitinib can restore cellular sensitivity to steroids, thereby improving response rates.^15^ Further, tofacitinib differs pharmacokinetically from other available rescue therapies. Time to peak serum concentration of tofacitinib ranges from 30 min to 1 h, and the half-life elimination is 3 h,^16^ considerably shorter than cyclosporin (peak serum concentration in 1.5-2 h and half-life 5-18 h) and infliximab (variable peak serum concentration depending on albumin and inflammatory burden, half-life 7-12 days).^17,18^ Tofacitinib is minimally bound to protein, thus does not depend on serum albumin, and is not affected by inflammatory burden to reach peak-concentration with minimal wasting. The short half-life of tofacitinib may also lower concern for cumulative effects of immunosuppression agents when considering a third-line rescue therapy. Indeed, in our trial, among 4 participants who did not respond to tofacitinib within the first 7 days of treatment and were treated with third-line medical rescue therapies (infliximab or upadacitinib), 3 demonstrated clinical response and were able to be discharged from hospital without complications.

The safety profile of tofacitinib has been under scrutiny due to potential concern for cardiovascular events, venous thromboembolism, and serious infections.^19^ Although an association between tofacitinib and venous thromboembolism/cardiovascular events was observed in those with rheumatoid arthritis and cardiovascular risk factors, this finding has not been observed in the ulcerative colitis population.^20^ A post hoc analyses of 5 studies that included 1157 UC patients with up to 6.8 years of treatment with tofacitinib demonstrated that age was a statistically significant predictor of adverse effects including herpes zoster, and malignancies.^21^ This analysis also showed that thrombotic events were infrequent in the tofacitinib group regardless of age, though the study population had few participants over the age of 65. In our study, 5 of 24 individuals experienced an adverse event. Most events were mild. One 37-year-old individual had a syncopal event followed by a stroke on day 3 of tofacitinib therapy. This event may have been caused by tofacitinib, but this individual had multiple comorbidities and thrombotic risk factors beyond ASUC, including HIV, recent cocaine use, and was critically ill with a high inflammatory burden eventually ending up in hemorrhagic shock, all of which may have been contributing factors for ischemic stroke. All patients treated in this trial also received pharmacologic prophylaxis against venous thromboembolism, consistent with ASUC guideline recommendations.^2^

This study has a number of strengths. Firstly, the participants all had failed first-line intravenous corticosteroid rescue therapy, and one-third had previously failed anti-TNF therapy. Conventional treatment paradigms would have recommended colectomy or cyclosporin for many of these patients with biologic failure, but this data suggests tofacitinib could also be an option. The TACOS trial demonstrated efficacy of tofacitinib as adjunctive therapy to corticosteroids, although this trial design differs as TRIUMPH studies patients with corticosteroid failure and includes those with prior biologic failure as well.^10^ Furthermore, the induction dose of tofacitinib used in this study is 10mg twice daily, which is the on label dose of tofacitinib in moderate-to-severe UC, and lower than prior studies that used off label 3 times daily dosing.^10,22^ Our study suggests that this lower induction regimen may be an option as well which is consequential as many of the adverse effects of tofacitinib are thought to be dose-related.^23^ The design of this trial also has inherent weakness. As it is an open-label design with no control group, there is potential for bias, including selection, performance, detection, attrition, and confirmation bias. At the time of recruitment, during the informed consent process, participants were counseled on alternative medical salvage therapies should they not respond to tofacitinib with the aim of minimizing performance and detection bias.

## Conclusion

In conclusion, tofacitinib 10 mg twice daily may be an effective rescue therapy in hospitalized patients with steroid-refractory ASUC. Tofacitinib is available in generic form in many jurisdictions so may become a preferred option for cost and convenience considerations as well. Further controlled studies are warranted to understand how to position tofacitinib, and other JAK inhibitors, relative to infliximab in the therapeutic algorithm of ASUC.

## Supplementary Material

otaf013_suppl_Supplementary_Figures

otaf013_suppl_Supplementary_Tables

## Data Availability

Data not publicly accessible but could be made available upon reasonable request to the study authors
