# Supplementary material for: Tofacitinib for Hospitalized Acute Severe Ulcerative Colitis Management (The TRIUMPH Study)
Source: Crohns Colitis 360. 2025 Feb 15;7(1):otaf013. doi: 10.1093/crocol/otaf013 (PMC11906967; doi:10.1093/crocol/otaf013)
Supplement: otaf013_suppl_Supplementary_Figures [file otaf013_suppl_supplementary_figures.zip › Supplementary Table and Figure Legend_Supplementary Figure 1_Supplementary Figure 2_Supplementary Figure 3/Supplementary Table and Figure Legend.docx]

Supplementary Table and Figure Legend

Supplementary Table 1 – Baseline characteristics stratified by day 7 clinical response status.

Supplementary Table 2 - Univariate analyses of baseline characteristics to predict day 7 clinical response.

Supplementary Table 3 - Adverse events experienced over the course of treatment of 52 weeks.

Supplementary Figure 1 - Percentage of responders and non-responders who achieved MTWSI diarrhea subscore of 0 or 1

Supplementary Figure 2 - Percentage of responders and non-responders who achieved MTWSI visible blood in stool subscore of 0 or 1

Supplementary Figure 3 - Daily median CRP trends for tofacitinib responders vs non-responders.
