# Supplementary figures and images for: Tofacitinib for Hospitalized Acute Severe Ulcerative Colitis Management (The TRIUMPH Study)
Source: Crohns Colitis 360. 2025 Feb 15;7(1):otaf013. doi: 10.1093/crocol/otaf013 (PMC11906967; doi:10.1093/crocol/otaf013)

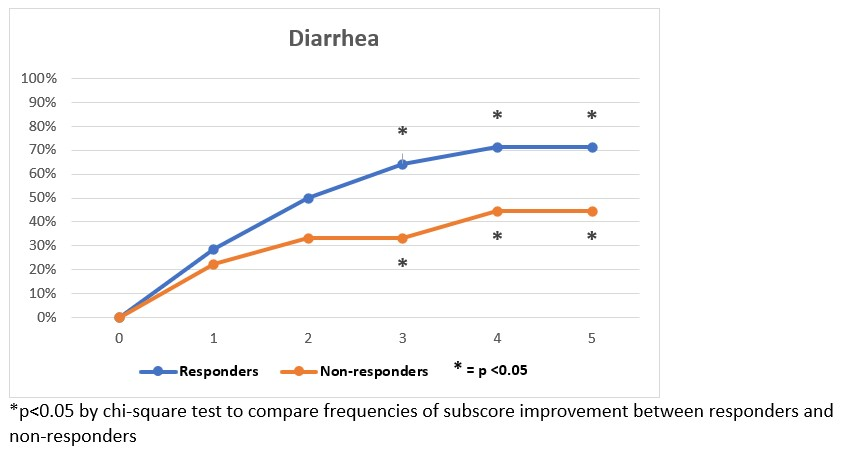

Supplement: otaf013_suppl_Supplementary_Figures [file otaf013_suppl_supplementary_figures.zip › Supplementary Table and Figure Legend_Supplementary Figure 1_Supplementary Figure 2_Supplementary Figure 3/Supplementary Figure 1.tiff]

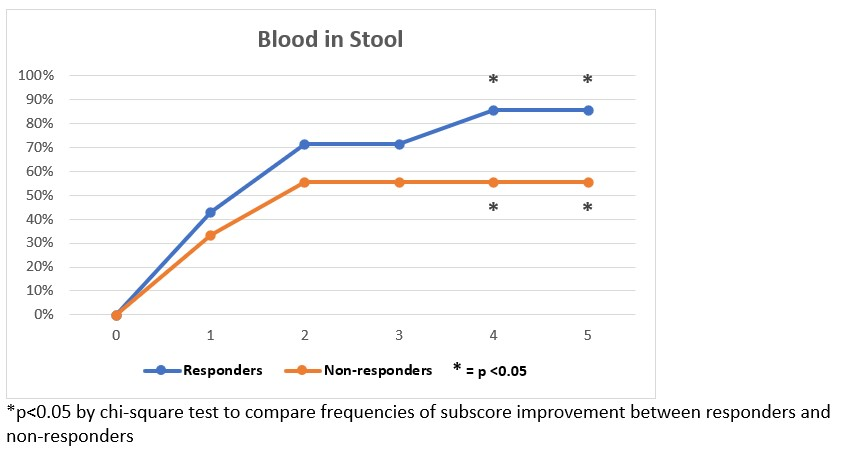

Supplement: otaf013_suppl_Supplementary_Figures [file otaf013_suppl_supplementary_figures.zip › Supplementary Table and Figure Legend_Supplementary Figure 1_Supplementary Figure 2_Supplementary Figure 3/Supplementary Figure 2.tiff]

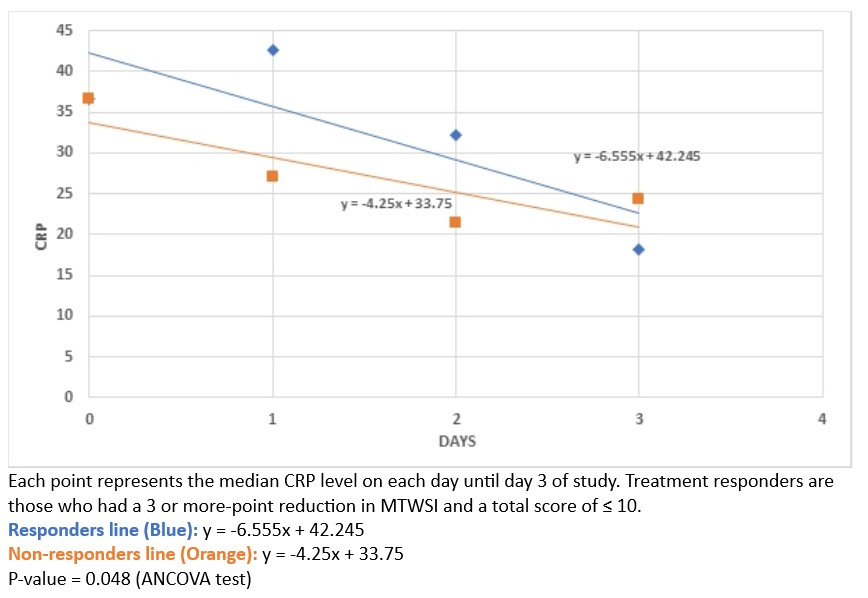

Supplement: otaf013_suppl_Supplementary_Figures [file otaf013_suppl_supplementary_figures.zip › Supplementary Table and Figure Legend_Supplementary Figure 1_Supplementary Figure 2_Supplementary Figure 3/Supplementary Figure 3.tiff]
