# Supplementary material for: Tofacitinib for Hospitalized Acute Severe Ulcerative Colitis Management (The TRIUMPH Study)
Source: Crohns Colitis 360. 2025 Feb 15;7(1):otaf013. doi: 10.1093/crocol/otaf013 (PMC11906967; doi:10.1093/crocol/otaf013)
Supplement: otaf013_suppl_Supplementary_Tables [file otaf013_suppl_supplementary_tables.docx]

**Supplementary Table 1 – Baseline characteristics stratified by day 7 clinical response status**

|  | **Responders** | | **Non-Responders** |
| --- | --- | --- | --- |
| **Number of Patients** | 14 | | 9 |
| **AGE, mean (SD)** | 41 (15.6) | | 46.5 (20.2) |
| **SEX** | Male | 7 | 5 |
|  | Female | 7 | 4 |
| **Disease Location** | Pancolitis | 2 | 2 |
|  | Left-sided | 10 | 6 |
|  | Extensive colitis | 2 | 1 |
| **Previous Biologics (%)** | 5 (35.71) | | 3 (33.3) |
| **Lab** | Hgb, mean (SD) | 100.4 (20.9) | 97.3 (20.1) |
|  | CRP, median (IQR) | 37.9 (50.5) | 36.6 (18.8) |
|  | Fecal Calprotectin, median (IQR) | 2,641(3238.6) | 2672 (4205.4) |
|  | Albumin, mean (SD) | 29.1 (5) | 27.6 (6) |
| **Mayo Endoscope subscore 3, (%)** | 10 (71.4) | | 7 (77.8) |
| **Total Mayo Score, mean (SD)** | 9.7(1.6) | | 10.6 (0.7) |

(Patient who withdrew after one day of treatment was excluded from this analysis)

**Supplementary Table 2 - Univariate analyses of baseline characteristics to predict day 7 clinical response**

| **Baseline variable** | **Univariable p-value** |
| --- | --- |
| AGE | 0.58 |
| SEX | 0.63 |
| DISEASE LOCATION | 0.36 |
| PREVIOUS BIOLOGICS | 0.66 |
| Hgb | 0.73 |
| CRP | 0.21 |
| FECAL CALPROTECTIN | 0.95 |
| ALBUMIN (=<30 vs >30) | 0.36 |
| ALBUMIN (CONTINUOUS) | 0.59 |
| MAYO ENDOSCOPIC SUBSCORE 3 | 0.61 |
| TOTAL MAYO SCORE (<10 vs >= 10) | 0.26 |
| TOTAL MAYO SCORE (CONTINUOUS) | 0.10 |

**Supplementary Table 3 - Adverse events experienced over the course of treatment of 52 weeks.**

|  |  | Adverse Event Type |
| --- | --- | --- |
| **Total Number of AE** | 13 | C. Difficile, Leukocytosis, Elevated Triglycerides, Left Eye Redness, Elevated CRP, Hand Tremors, UC Flare, Headache, Tachycardia, Nausea, Decreased Appetite, Stroke, Syncope |
| **Total Number of patients with AE** | 5 |  |
| **Total number of serious AE** | 1 | Stroke |
